# NMR screening of low molecular weight inhibitors targeting the papain‐like protease (PLPro) of SARS‐CoV‐2

**DOI:** 10.1002/2211-5463.70082

**Published:** 2025-07-01

**Authors:** Dennis J. Pyper, Sridhar Sreeramulu, Benjamin T. Lanham, Elizabeth M. Engle, David Fushman, Harald Schwalbe

**Affiliations:** ^1^ Institute of Organic Chemistry and Chemical Biology, Center for Biomolecular Magnetic Resonance (BMRZ) Goethe‐University Frankfurt/M Germany; ^2^ Center for Biomolecular Structure and Organization, Department of Chemistry and Biochemistry University of Maryland College Park MD USA

**Keywords:** coronavirus, fragment‐based NMR screening, NMR spectroscopy, Nsp3d, papain‐like protease PLPro, SARS‐CoV‐2

## Abstract

The Papain‐like protease (PLPro) from SARS‐CoV‐2 plays an important role in the cleavage of the polyproteins Pp1a and Pp1ab as well as in the suppression of the immune response by deISG15ylation. Considerable effort is therefore devoted to developing low molecular weight inhibitors as starting points for antiviral drugs. Here, we present the results of an NMR screening study of PLPro for binding to the DSI‐poised fragment library containing 607 compounds. Based on saturation‐transfer difference (STD)‐ and WaterLOGSY‐NMR experiments, we identified 86 binding compounds. We prioritized five candidates for further in‐depth analysis. For three of those, we determined dissociation constants and two distinct binding sites on PLPro. These compounds could serve as a basis for future drug design studies in medicinal chemistry.

## Graphical abstract

Papain‐like protease from SARS‐CoV‐2 plays an important role in the cleavage of the viral polyproteins and in the suppression of the host's immune response. Here, we present the results of an NMR screening study. We identified 86 binding compounds, of which five candidates were chosen for in‐depth analysis. For three, we determined dissociation constants and two distinct binding sites.

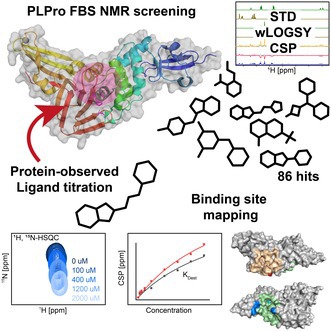

**Abbreviations**
Nsp: nonstructural protein
PLPro: papain‐like protease
SARS‐CoV‐2: severe acute respiratory syndrome coronavirus type 2
STD: saturation‐transfer difference
WLOGSY: WaterLOGSY

The global pandemic caused by the SARS‐CoV‐2 virus has been responsible for more than 7 million confirmed deaths worldwide, and associated deaths are estimated to be even higher than 20 million [1]. Research is ongoing for the development of antiviral inhibitors as starting points for the development of future therapeutics [2]. The genome of SARS‐CoV‐2 codes for two viral proteases: MPro and PLPro which catalyze the cleavage of the viral polyprotein chains Pp1a and Pp1ab into 16 different nonstructural proteins [3]. PLPro is one of eight domains of the 1945 amino acid long nonstructural protein 3 (Nsp3), starting at position 746. It consists of 315 amino acids. PLPro cleaves after Nsp1, Nsp2, and Nsp3, recognizing a C‐terminal LXGG motif and thus releasing these proteins as well as itself [4]. The protease carries also deubiquitinating and deISG15ylating enzymatic activities, which can disrupt the antiviral immune system response of the host [5]. Furthermore, it amplifies NF‐κB signaling contributing to a burst of cytokine release which is partially responsible for the severe effects of COVID‐19 infections [6].

Structurally, PLPro has a thumb–palm–fingers architecture with an N‐terminal ubiquitin‐like domain at the thumb part (Fig. 1). The catalytic site is located between the palm and the thumb. It contains the three amino acids C111, H272, and D286 forming a cysteine protease catalytic triad. A Zn^2+^‐binding site consisting of four cysteines is located in the fingers domain. This Zn^2+^‐binding site has been shown to be important for structural stability and proteolytic activities. Within close proximity of the three amino acids that form the catalytic triad, four binding pockets are localized labeled S1 through S4 as well as an important loop (labeled BL2 loop) that can close upon binding of substrates [7]. Low molecular weight compounds (small molecules) that inhibit the catalytic activity of PLPro bind mostly in the S3/S4 pockets blocking access to the active site [8]. Furthermore, the S4 pocket is responsible for the cleavage motif recognition (LXGG) by recognizing its leucine residue. The other amino acids of the LXGG motif fit into the other binding pockets, S3 to S1, placing the C‐terminal residues right at the catalytic center. Thus, it facilitates the cleavage of ubiquitin and ISG15 (both contain C‐terminal LRGG motif) as well as the first three viral polyproteins [9].

**Fig. 1:**
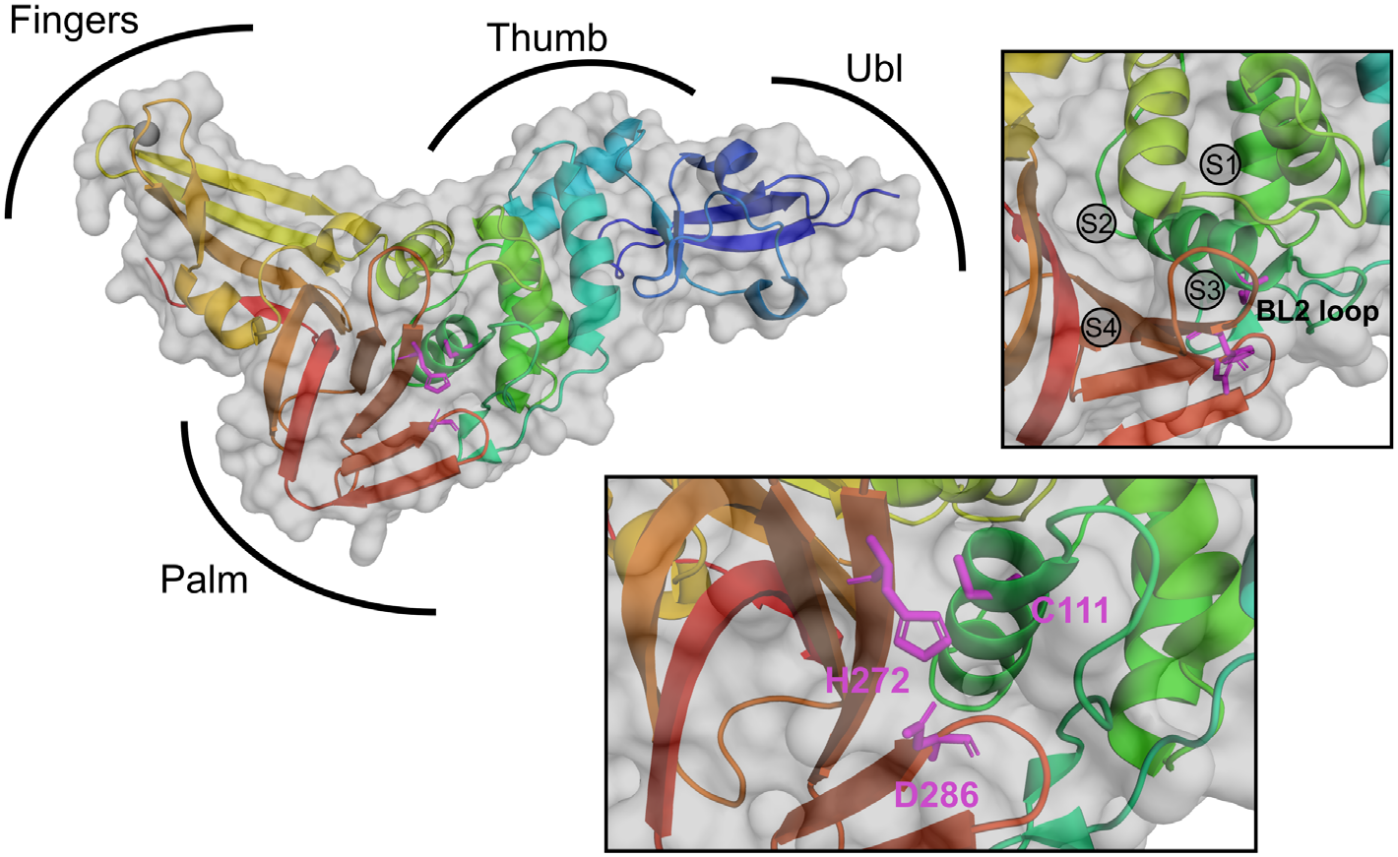
Crystal structure of PLPro (6WZU) with the regions of the thumb–palm–fingers architecture as well as the ubiquitin‐like domain marked. On the top right, the zoomed‐in structure is shown indicating the positions of the binding pockets and the BL2 loop. On the bottom, the section of the active site is zoomed in to show the position of the catalytic triad.

The search for PLPro inhibitors started with revisiting the known inhibitors of SARS‐CoV‐1 PLPro. The two proteases have a sequence identity of 83% and are structurally highly similar, including the active site [7]. An example of an inhibitor of SARS‐CoV‐1 PLPro is GRL0617 that binds non‐covalently in the S3 and S4 binding pockets of the protein and inhibits its ability to cleave the viral polyproteins and to deubiquitinate and deISG15ylate host proteins [10]. This compound has also been heavily modified to produce a plethora of derivatives with the goal of improving the binding affinity as well as increasing its inhibitory activity [8].

In order to identify additional starting points for medicinal chemistry campaigns, we report the results of an NMR spectroscopy fragment‐based screening using the DSI‐poised library with 607 compounds [11]. We found 86 binding fragments to exhibit a positive STD and/or WLOGSY effects, indicating binding to PLPro. We chose five promising compounds for an in‐depth analysis by protein‐observed ligand titration experiments. We were able to estimate the *K*_*D*_ and to map the CSPs onto an existing crystal structure, indicating that next to demonstrating orthosteric binding of the fragments to the active site, the ligands also bind to an alternative site and allow to selectively address the inhibition of the protease activity versus the deubiquitination activity of PLPro.

## Materials and methods

The PLPro construct that was used in this study carried two point mutations: C111S and K232Q. The protein was heterologously expressed in *E. coli* BL21 (DE3) gold cells. Protein expression and purification has been described before [12, 13].

Fragment‐based NMR screening and sample preparation for screening have been described before [14, 15]. The fragments were premixed in batches of four fragments with a concentration of 12.5 mm per fragment in d_6_‐DMSO with 10% D_2_O. The fragment mixes were pipetted onto two 96‐well plates. The protein solution was added to each mix for a final concentration of 10 μm protein and 25 μm fragment mixture. 35 μL of these mixtures were then transferred to 1.7 mm NMR tubes and DSS was added for a concentration of 5 μm as an internal reference. The final DMSO concentration amounted to 4.5%. The protein buffer contained 25 mm BisTris pH 7.4, 100 mm NaCl, 1 mm TCEP, and 1 μm ZnCl_2_. The samples were prepared using a pipetting robot. Measurements were carried out at 298 K on a 600 MHz Bruker spectrometer equipped with a 1.7 mm Cryo TCI 1H [^13^C, ^15^N] probehead (spectral acquisition parameters shown in Table S1).

Ligand titrations were carried out by preparation of individual samples for every step, keeping constant concentrations of protein (50 μm) and DMSO (4% (v/v)). The titration steps were at [ligand] = 50, 100, 200, 400, 800, 1200, 1600, and 2000 μm. For each titration step, ^1^H 1D and ^1^H‐^15^N BEST‐TROSY [16, 17, 18, 19] spectra were measured at 20 °C. In addition, a blank sample containing only protein and DMSO was measured before and after the titration measurements. All measurements were carried out on a 600 MHz spectrometer equipped with a 1.7 mm Cryo TCI 1H [^13^C, ^15^N] probehead (spectral acquisition parameters shown in Table S1).

It was checked that the fold of the protein is retained even at buffer conditions of 4% DMSO (Fig. S1).

CSPs were calculated using the following formula:

$$\Delta \delta =\sqrt{0.14{\left(\Delta \delta_{N}\right)}^{2}+{\left(\Delta \delta_{H}\right)}^{2}}$$

The dissociation constants *K*_*D*_ were estimated by plotting the CSPs against the ligand concentration and fitting them using the following formula derived from [20, 21]:

$$\Delta \delta_{\mathrm{obs}}=\begin{array}{l} \Delta \delta_{\max} \end{array}\left({\left[P\right]}_{0}+{\left[L\right]}_{0}+K_{D}-\sqrt{{\left({\left[P\right]}_{0}+{\left[L\right]}_{0}+K_{D}\right)}^{2}-4{\left[P\right]}_{0}{\left[L\right]}_{0}}\right)/2{\left[P\right]}_{0}$$

where [P]_0_ and [L]_0_ are the total concentrations of the protein and ligand, respectively, at a given titration point, and Δ*δ*_max_ is the CSP value at full saturation.

## Results and discussion

We chose a PLPro construct containing two amino acid point mutants, namely C111S and K232Q, which help in stabilizing the protein against rapid precipitation. C111S mutates the active cysteine of the catalytic triad. Amino acid 232 is a glutamine in the SARS‐CoV‐1 variant of PLPro as well as the delta variant of SARS‐CoV‐2. Crystal structures of the C111S mutant show that the mutation does not alter the PLPro structure in a significant way [7]. Furthermore, it has been shown in crystal structures that known binders interact also with the active site mutant [6, 13, 22].

**Fig. 2:**
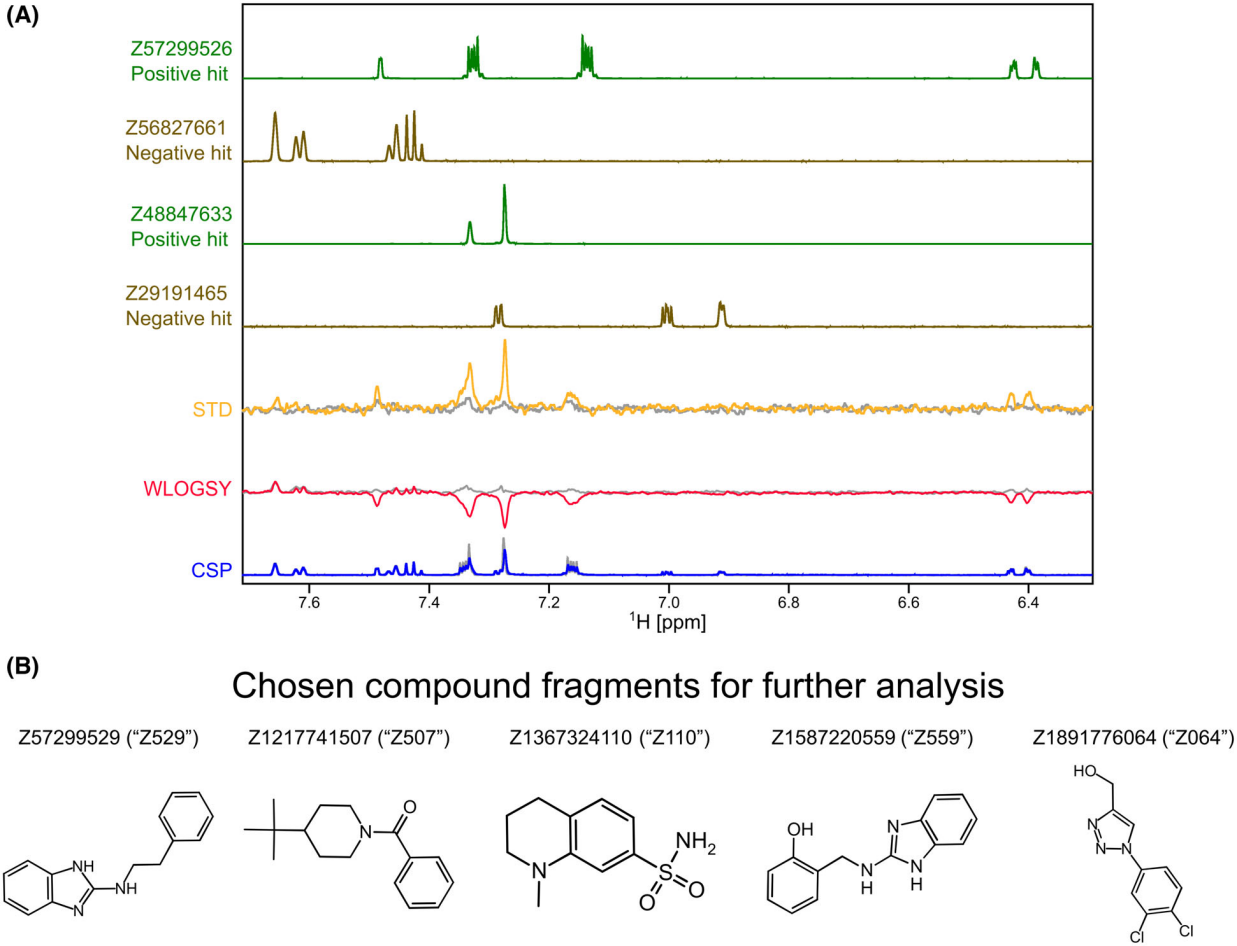
(A) An example for the analysis of two initial screening hits. The first four 1D ^1^H spectra correspond to the isolated fragment compounds. Below are the STD (yellow) and WLOGSY (red) spectra showing changes upon interaction with the protein in comparison to no added protein (gray). On the bottom, a standard 1D spectrum (blue) with and without protein (gray) is shown to analyze CSPs and line broadening. (B) Five compounds were chosen from the initial screening for further analysis. The compounds and their structural formulas are shown.

The screening of the DSI‐poised library against PLPro involved the measurement of 1D ^1^H experiments for CSP and line broadening analysis as well as STD and waterLOGSY (WLOGSY) for sensitive detection of binding. In the case of STD, a binding compound will produce signals in the STD spectrum similar to its signals in a normal 1D spectrum, whereas a non‐binding compound will have no signals. In the WLOGSY spectrum, signals appear for both binders and non‐binders. However, signals from binders will be negative. CSP, STD, and WLOGSY were used in conjunction for the identification of possible binding fragments. 86 hits were identified showing a signal in the STD spectra, a WLOGSY sign change, or both. No fragments showed significant CSPs or line broadening in the 1D spectra. Our initial hits are shown in Table S2. An example for the analysis of two positive hits is shown in Fig. 2A. We then narrowed the selection of compounds down for follow‐up experiments employing two different prioritization strategies: We first narrowed down the number of follow‐up studies to 34 compounds by choosing hits that had both an STD signal and a WLOGSY sign change in at least one signal in the 1D NMR spectrum (compounds shown in Fig. S2). Second, we further reduced that number to twelve by choosing compounds that fulfilled our requirements for all of their aromatic and aliphatic signals with high signal‐to‐noise ratios. Of those twelve, five candidates were then chosen randomly for further investigations: Z57299529 (“Z529”), Z1217741507 (“Z507”), Z1367324110 (“Z110”), Z1587220559 (“Z559”), and Z1891776064 (“Z064”) (shown in Fig. 2B). Additionally, they are quite chemically diverse (low Tanimoto coefficient, see Table 1) with the exception of Z529 and Z559. All investigated compounds also share low similarities with the well‐characterized PLPro inhibitor GRL0617 (also shown in Table 1).

**Table 1:** Tanimoto coefficients of the five ligands chosen for in‐depth analysis. With the exception of Z529 and Z559, the coefficients are quite low, indicating chemical diversity. Additionally, the Tanimoto coefficients of the five ligands in comparison with GRL0617 are also shown.

	Z57299529	Z1217741507	Z1367324110	Z1587220559	Z1891776064	GRL0617
Z57299529	1	0.09	0.048	0.446	0.075	0.071
Z1217741507	0.09	1	0.11	0.087	0.082	0.101
Z1367324110	0.048	0.11	1	0.047	0.079	0.074
Z1587220559	0.446	0.087	0.047	1	0.086	0.081
Z1891776064	0.075	0.082	0.079	0.086	1	0.053
GRL0617	0.071	0.101	0.074	0.081	0.053	1

Protein‐based ^1^H‐^15^N BEST‐TROSY spectra were measured for eight titration steps and assigned using the published assignment by Shiraishi and Shimada [23]. We also assigned the backbone of the protein independently using a uniformly deuterated ^13^C^15^N‐labeled sample. While our assignment did not exceed the published assignment, we were able to confirm it. Following the binding by detecting CSPs of the protein target was severely hampered due to substantial spectral overlap. Compound Z507 showed no CSPs in the protein‐based BEST‐TROSY spectrum despite showing clear STD and WLOGSY effects in the ligand‐based screening. Z064 showed CSPs at lower ligand concentrations. However, around 200 μm of ligand, the protein started precipitating, lowering the signal‐to‐noise in the spectra as well as the tubes being visibly cloudy. Further analysis of this compound was therefore not feasible. Z529 proved to be a high‐quality fragment. We here focus on data for the Z529 hit due to clear protein CSPs. Similar analyses for Z110 and Z559 (medium‐quality) can be found in the supplementary information (Figs S3 and S4).

**Fig. 3:**
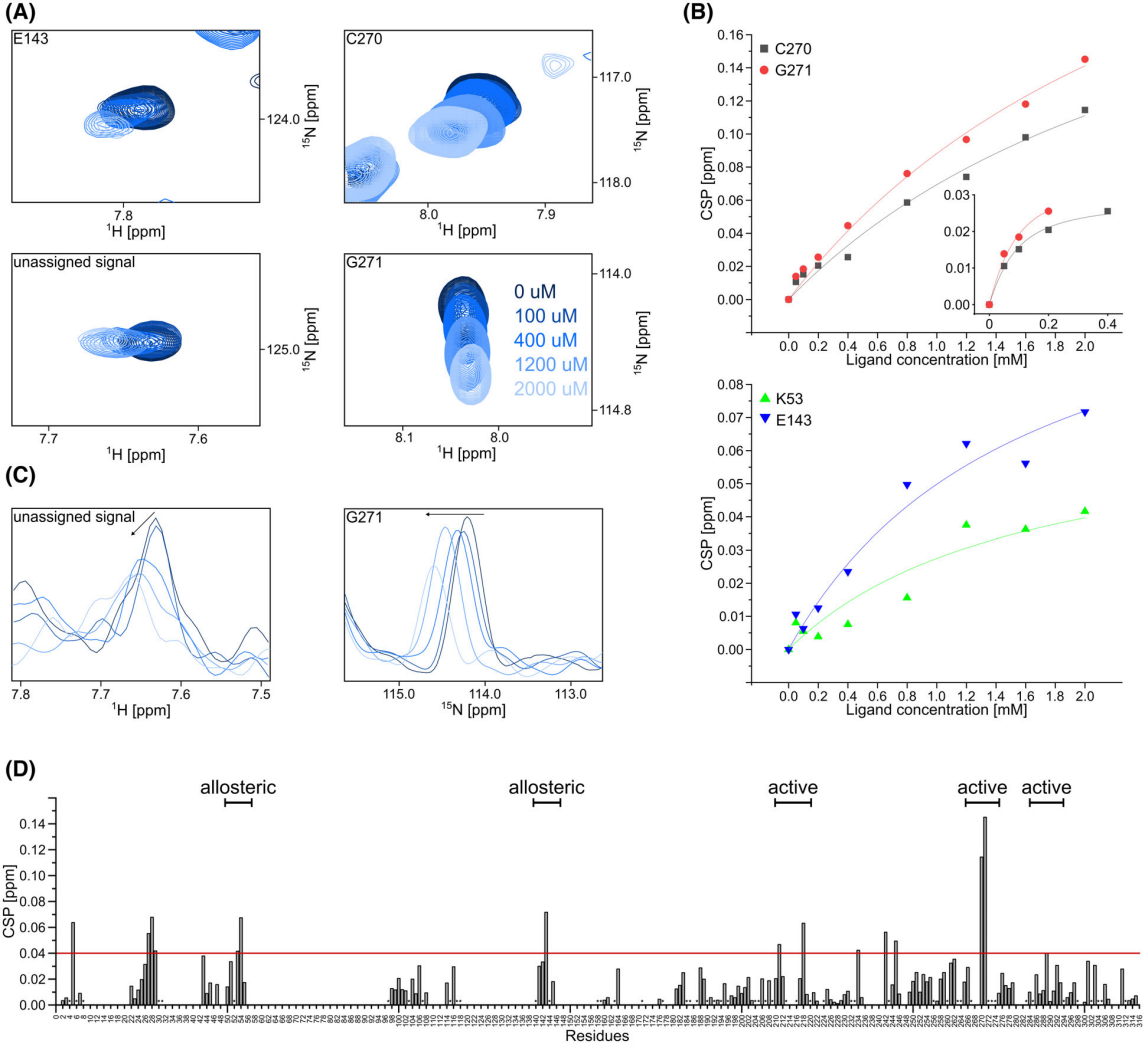
Analysis of compound Z529. (A) Four examples of shifting signals from the titration. Included signals are of residues E143, C270, G271 and an unassigned signal. Included titration steps are 0, 100, 400, 1200 and 2000 μm. (B) CSPs at rising ligand concentrations plotted for selected amino acids and fitted for *K*_*D*_ estimation. The first plot shows the global fit for the CSPs of C270 and G271 that are part of the BL2‐loop and thus show the binding of the compound to the active site. The first four data points for C270 and the first three data points for G271 are also plotted and globally fitted separately (see inset), as they seem to follow a different curve. This could suggest two binding events. The second plot shows the amino acids K53 and E143 which are part of the potential alternative binding site. Estimated *K*_*D*_'s are 3.0 ± 0.7 mm for the active site and 1.5 ± 0.6 mm for the alternative site. If only the first four/three data points for C270/G271 are fitted, an estimated *K*_*D*_ of 0.06 ± 0.01 mm is determined. (C) 1D projections of the unassigned signal and of G271 signal. For the unassigned signal, the signal shifts in the ^1^H dimension and its intensity decreases. The signal of G271 shifts mainly in the ^15^N dimension. This indicates different exchange modes for the two signals. (D) Bar plot of CSPs at 2000 μm for every residue. Stars indicate that the amino acid has an assignment but could not be plotted due to, for example, overlap with other signals. On top, it is indicated which CSPs belong to which binding site.

Z529 shows various signal shifts (Fig. 3D), most prominently for C270 and G271, which are both part of the BL2‐loop. Additionally, T4, I5, Y27, G28, Q29, K53, T54, E143, N177, L211, K218, L234, V242, S245, and L289 show slightly lower CSPs. Furthermore, we found various signals that show CSPs that were not included as no assignment for those signals was available. Examples can be found in Fig. 3A. There also seem to be two different exchange modes as seen in Fig. 3C: the unassigned signal shifts in the ^1^H dimension and loses intensity, while the signal for G271 mostly shifts in the ^15^N dimension with the same intensity except for the last titration point. The CSPs were plotted against the concentration of the ligands and globally fitted to estimate the dissociation constants (shown in Fig. 3B). For this, a distinction was made between CSPs in the active site and CSPs near the alternative site, and they were plotted separately from each other. The *K*_*D*_ for the active site (gained from C270 and G271) was estimated to be 3.0 ± 0.7 mm; the *K*_*D*_ for the alternative site (K53 and E143) was estimated to be 1.5 ± 0.6 mm. The second *K*_*D*_ is slightly lower, which could indicate that the compound will preferentially bind to the alternative binding site. The data were conservatively fitted over all data points. However, the shape of the curve for the active site suggests two binding events, with different affinities. Figure 2B shows an additional fit for the CSPs of C270 and G271: only the first four data points for C270 and the first three for G271 were included in the global fit, resulting in an estimated *K*_*D*_ of 0.06 ± 0.01 mm. Furthermore, this could explain the relatively high number of initial hits in the screening due to multiple available binding sites in the protein.

**Fig. 4:**
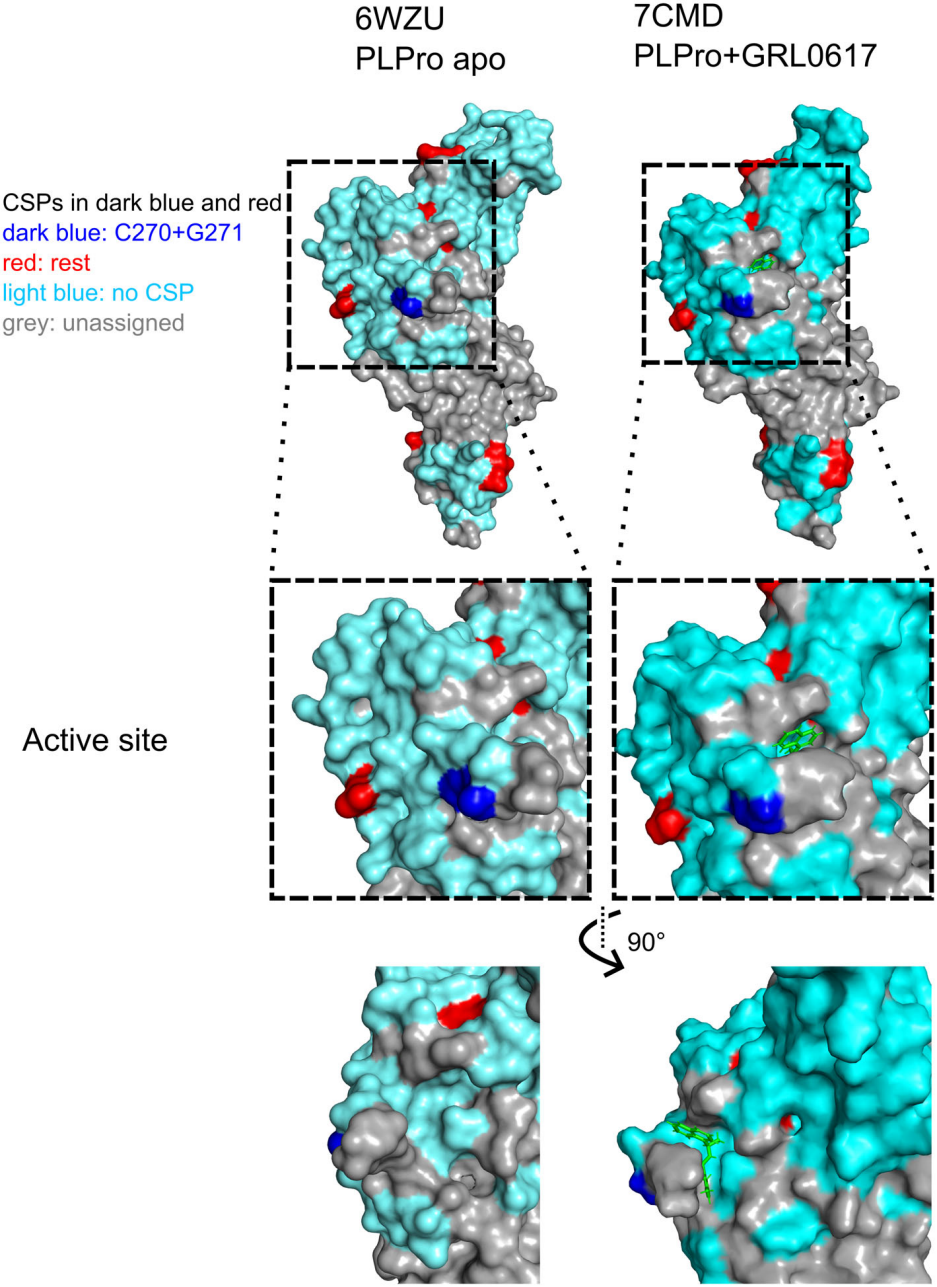
Mapping of CSPs from Z529 titration onto the crystal structure of PLPro (6WZU). On the right, the crystal structure of PLPro + GRL0617 is shown for comparison. Several CSPs are located near the binding sites, indicating that Z529 would be similarly bound to PLPro as GRL0617 is. CSPs are colored in dark blue (C270 + G271) and red (other amino acids). Everything that was assigned in the NMR spectra is colored light blue. Missing assignments are in gray.

All CSPs above 0.04 ppm (standard deviation of all CSPs at 2 mm ligand) were mapped onto the existing crystal structure of PLPro (PDB: 6WZU [7]) shown in Fig. 4. Additionally, on the right, the crystal structure of PLPro with the known binder GRL0617 (PDB: 7CMD [24]) is shown. Judging from the recorded CSPs caused by Z529, notably C270 and G271 in dark blue, it is likely that the fragment is binding in the immediate proximity of the active site in a similar fashion to GRL0617.

In order to further validate our data, we performed an FTMap [25] analysis of PLPro (6WZU). FTmap scans the surface area of the protein using 16 different extremely small chemical probes, for example, acetone, ethanol, or urea. If several of these probes are found to bind to the same spot, a cross‐cluster is formed. Comparison of the predicted binding spots of the chemical probes with the partial experimental identification of binding sites of the three ligands of interest was combined. We integrated the cross‐clusters with a cleft analysis from PDBsum [26].

**Fig. 5:**
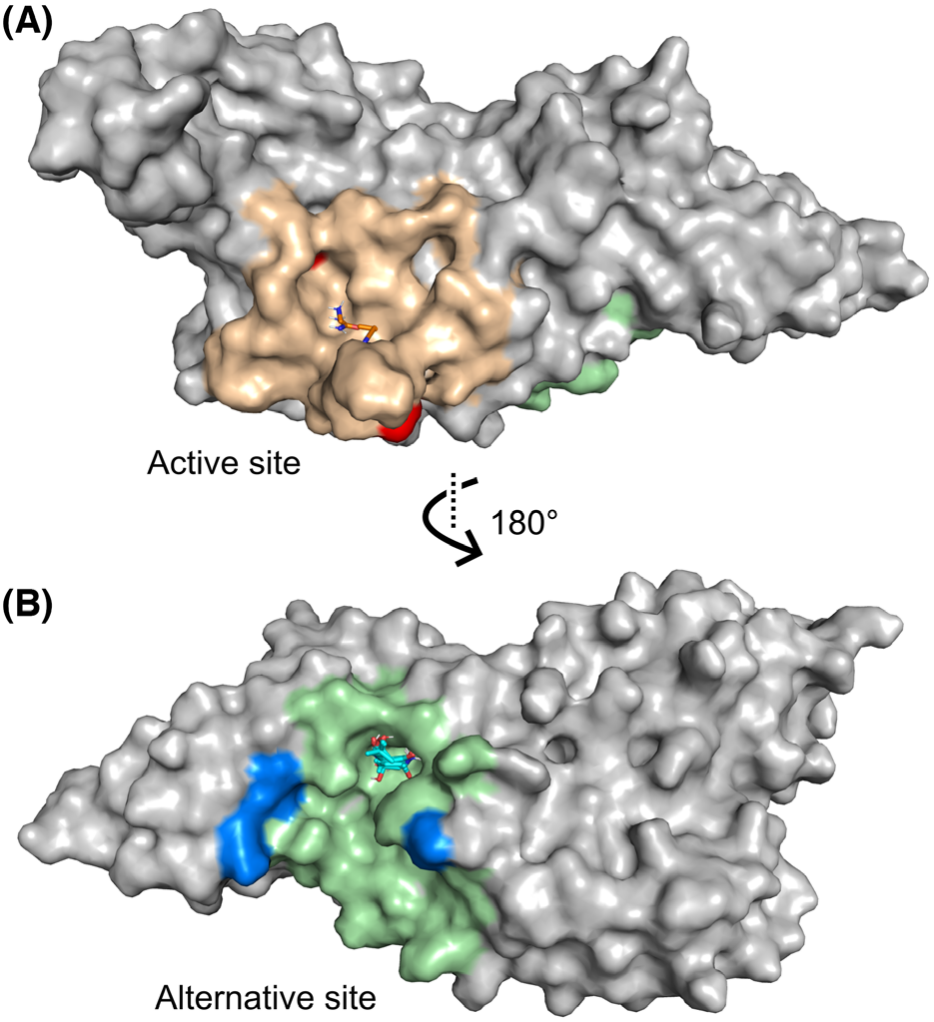
The results of FTMap and PDBsum analyses. The clefts calculated by PDBsum are colored in beige for the active site and green for the alternative site. Relevant CSPs within the clefts are colored in red and blue. The cross‐clusters obtained by the FTMap analysis are shown inside the clefts to indicate the possible binding sites of ligands. A shows the cleft and cross‐cluster for the active site, B shows the alternative site on the other side of the protein.

This procedure showed two reasonable binding sites for Z529: the already known and well‐established binding in the active site as well as an alternative site on the other side of the protein which, interestingly, has also been previously identified by Ferreira et al. [27] Another cleft from the PDBsum that aligns with three cross‐clusters from FTMap is shown in Fig. S5, which is also referenced by Ferreira et al. The relevant CSPs in this area, however, were not feasible for *K_D_* determination and binding site validation. Figure 5A shows one cross‐cluster (in beige) of small molecules inside a cleft with experimental CSPs from Z529 titration highlighted in red. Figure 5B shows the alternative site. Notably, most of the cleft of the alternative binding site (in green) was not assigned in the spectra; thus, only a few CSPs (highlighted in blue) could be recorded there. As seen in Fig. 5, the alternative site is remote from the active site.

## Similarity analysis

All five compounds identified in the NMR screening were compared to known binders in a similarity analysis. We calculated the Tanimoto coefficient of our compounds and for every binder that has been co‐crystallized with PLPro, from the PDB. The most similar compounds for every fragment hit are shown in Table 2 (full results in Table S3). The binding pocket for all compounds is the active site of the protein, which corresponds to our findings of CSPs being primarily in this region. The highest Tanimoto coefficient does not exceed 0.172, which is low, and indicates that the NMR screening campaign identified novel compounds compared to previous structural biology‐supported screening campaigns.

**Table 2:** Overview of the calculated Tanimoto coefficients between our screening compounds and known PLPro binders from the PDB. Only the compounds with the highest coefficients are shown. The full results can be found in Table S3.

Screening compound		PDB compound		Tanimoto coefficient
Z57299529	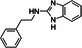	Ebselen^28^	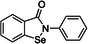	0.139
Z1217741507	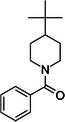	Compound S43^29^/ebselen	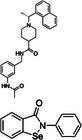	0.172
Z1367324110	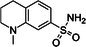	Remodilin NCGC 390004^30^	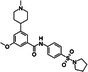	0.162
Z1587220559	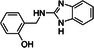	Jun9‐84‐3^31^	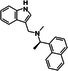	0.14
Z1891776064	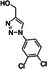	4‐(2‐hydroxyethyl)phenol^32^	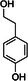	0.127

## Conclusion

Research into SARS‐CoV‐2 still remains relevant. New inhibitors of different viral proteins are identified and present possibilities for new drugs. After SARS and MERS SARS‐CoV‐2 is the third coronavirus pandemic of this century. Initial development of compounds targeting SARS‐CoV‐2 components was built on earlier research from those other pandemics, making it clear that the current research will also be highly relevant for a potential next Coronavirus‐related pandemic.

The screening of the DSI‐poised fragment library has revealed promising candidates that effectively bind to PLPro from SARS‐CoV‐2 and may inhibit its cleavage and anti‐immune response abilities. We found 86 fragment compounds in an initial NMR screen that showed WLOGSY and STD effects. For five of them, we performed protein‐observed ligand titrations, revealing low millimolar dissociation constants for three of them. Fragment Z529 seems to be the most promising with strong CSPs in the BL2‐loop in the vicinity of the active site of PLPro and a *K*_*D*_ of 3.0 ± 0.7 mm. The fragment has a good druglikeness‐score of 1.3 (calculated by DataWarrior [28]), a cLogP of 3.1, and several hydrogen‐acceptors and donors [29].

In addition, we identified a possible alternative binding site of the protein by comparing our additional CSPs to results from FTMap and PDBsum analysis. Alternative binding to the protein can be of interest for the development of new inhibitors. Additionally, a different fragment‐based screening study that was recently published [30] found that their identified hits also bind to either the active site or an alternative site. However, their identified alternative site is in the fingers‐region near the zinc‐finger binding site. Further research into binders to alternative sites of PLPro may be beneficial. Since the nature of our screening was to find fragments that effectively bind to the target protein, the compounds we identified can serve as a basis for future drug design studies in medicinal chemistry.

## Conflict of interest

We have no conflicts of interest to declare.

## Author contributions

DP: experiments, data analysis and interpretation, writing (original draft), writing (editing). SS: data interpretation, writing (editing). BTL: stability studies of PLPro variants, writing (editing). EME: stability studies of PLPro variants. DF: data interpretation, writing (editing). HS: funding acquisition, supervision, data interpretation, writing (original draft), writing (editing).

## Supporting information


**Fig. S1.** Superposition of two BEST‐TROSY spectra of PLPro with and without 4% DMSO in the buffer shown in red and black, respectively. Field strength varied between the two measurements: The spectrum with DMSO was measured at 600 MHz, the spectrum without DMSO was measured at 800 MHz. Protein concentration was sevenfold higher for the spectrum without DMSO (350 μm). The majority of the signals experience no or very minor shifts, meaning the fold of the protein is retained. Loss of low intensity signals in the spectrum with DMSO is due to the difference in protein concentration and field strength difference.
**Fig. S2.** Molecular structures and identifiers of all 34 ligands that showed both WLOGSY and STD effects in the fragment‐based screening.
**Fig. S3.** Analysis of compound Z1367324110. (A) Two examples of shifting signals from the titration. Included signals are of residues C270 and G271. Included titration steps are 0, 100, 400, 1200 and 2000 μm. (B) CSPs at rising ligand concentrations plotted for selected amino acids and globally fitted for *K*_*D*_ estimation (7.4 ± 6.5 mm). (C) Bar plot of CSPs at 2000 μm for every residues. Stars indicate that the amino acid has an assignment but could not be plotted due to e.g. overlap with other signals.
**Fig. S4.** Analysis of compound Z1587220559. (A) Two examples of shifting signals from the titration. Included signals are of residues C270 and G271. Included titration steps are 0, 100, 400, 1200 and 2000 μm. (B) CSPs at rising ligand concentrations plotted for selected amino acids and globally fitted for *K*_*D*_ estimation (2.1 ± 1.1 mm). (C) Bar plot of CSPs at 2000 μm for every residues. Stars indicate that the amino acid has an assignment but could not be plotted due to e.g. overlap with other signals.
**Fig. S5.** Additional cleft calculated by PDBsum shown in yellow on top of the protein. Relevant CSPs within the clefts are colored in red. Three cross‐clusters obtained by the FTMap analysis are shown inside the cleft to indicate the possible binding sites of ligands.
**Table S1.** Acquisition parameters for the NMR experiments of the fragment‐based screening and the protein‐observed ligand titrations.
**Table S2.** List of all 86 ligand hits from the initial fragment‐based screening.
**Table S3.** All Tanimoto coefficients between the five chosen screening compounds and known PLPro binders from the PDB.

## Data Availability

The data that support the findings of this study are openly available in GUDe at https://doi.org/10.25716/gude.1cp4‐6yyt, reference number gude.1cp4‐6yyt.
