# Supplementary material for: NMR screening of low molecular weight inhibitors targeting the papain‐like protease (PLPro) of SARS‐CoV‐2
Source: FEBS Open Bio. 2025 Jul 1;15(10):1667–77. doi: 10.1002/2211-5463.70082 (PMC12485815; doi:10.1002/2211-5463.70082)
Supplement: Supplementary file 1 — Fig. S1. Superposition of two BEST‐TROSY spectra of PLPro with and without 4% DMSO in the buffer shown in red and black, respectively. Field strength varied between the two measurements: The spectrum with DMSO was measured at 600 MHz, the spectrum without DMSO was measured at 800 MHz. Protein concentration was sevenfold higher for the spectrum without DMSO (350 μm). The majority of the signals experience no or very minor shifts, meaning the fold of the protein is retained. Loss of low intensity signals in the spectrum with DMSO is due to the difference in protein concentration and field strength difference. Fig. S2. Molecular structures and identifiers of all 34 ligands that showed both WLOGSY and STD effects in the fragment‐based screening. Fig. S3. Analysis of compound Z1367324110. (A) Two examples of shifting signals from the titration. Included signals are of residues C270 and G271. Included titration steps are 0, 100, 400, 1200 and 2000 μm. (B) CSPs at rising ligand concentrations plotted for selected amino acids and globally fitted for K D estimation (7.4 ± 6.5 mm). (C) Bar plot of CSPs at 2000 μm for every residues. Stars indicate that the amino acid has an assignment but could not be plotted due to e.g. overlap with other signals. Fig. S4. Analysis of compound Z1587220559. (A) Two examples of shifting signals from the titration. Included signals are of residues C270 and G271. Included titration steps are 0, 100, 400, 1200 and 2000 μm. (B) CSPs at rising ligand concentrations plotted for selected amino acids and globally fitted for K D estimation (2.1 ± 1.1 mm). (C) Bar plot of CSPs at 2000 μm for every residues. Stars indicate that the amino acid has an assignment but could not be plotted due to e.g. overlap with other signals. Fig. S5. Additional cleft calculated by PDBsum shown in yellow on top of the protein. Relevant CSPs within the clefts are colored in red. Three cross‐clusters obtained by the FTMap analysis are shown inside the cleft to indicate the p [file FEB4-15-1667-s001.docx]

**Supplementary information**

Supplementary table 1: Acquisition parameters for the NMR experiments of the fragment-based screening and the protein-observed ligand titrations.

|  | 1D 1H (FBS) | WLOGSY | STD | 1D 1H (titration) | BEST-TROSY |
| --- | --- | --- | --- | --- | --- |
| Number of scans | 128 | 256 | 128 | 128 | 128 |
| Resolution [Hz] | 1 | 1 | 1 | 1 | 14.29 (ω1); 20.92 (ω2) |
| Spectral width [ppm] | 16.03 | 16.03 | 16.03 | 16.03 | 15.15 (ω1); 33.03 (ω2) |
| TD | 19230 | 19230 | 19230 | 19230 | 1272 (ω1); 192 (ω2) |
| d1 [s] | 1 | 1 | 2 | 1 | 0.3 |
| Spectrometer frequency [MHz] | 600 | 600 | 600 | 600 | 600 |

Supplementary table 2: List of all 86 ligand hits from the initial fragment-based screening.

| **Ligand ID** | Molecular Formula | Molecular Mass [g/mol] | SMILES Code |
| --- | --- | --- | --- |
| Z100642432 | C9H9NOBrF | 246.08 | CN(C)C(c(ccc(F)c1)c1Br)=O |
| Z102895082 | C10H10N2O3 | 206.20 | COc1ccc(C=NNC2=O)c2c1OC |
| Z109092588 | C10H11NO3S | 225.27 | COC(c([s]cc1)c1NC(C1CC1)=O)=O |
| Z1152242726 | C11H11N3O2S | 249.29 | COc(cc1)ncc1NC(Nc1ccc[s]1)=O |
| Z1217741507 | C16H23NO | 245.37 | CC(C)(C)C(CC1)CCN1C(c1ccccc1)=O |
| Z1230032143 | C10H14N2O3S | 242.30 | CCOC(c1c(C)nc(N(C)C(C)=O)[s]1)=O |
| Z1250132544 | C9H11O2F | 170.19 | CC(COc1cccc(F)c1)O |
| Z1267773786 | C7H11N3 | 137.19 | CCNc1ccc(C)nn1 |
| Z1324080698 | C7H8NO2FS | 189.21 | Cc1cc(S(N)(=O)=O)cc(F)c1 |
| Z1359419878 | C9H8N2O2 | 176.18 | COc(cccc1)c1-c1nnc[o]1 |
| Z1367324110 | C10H14N2O2S | 226.30 | CN(CCC1)c2c1ccc(S(N)(=O)=O)c2 |
| Z1374778753 | C9H8N3OCl | 209.64 | OCc1c[n](-c(cc2)ccc2Cl)nn1 |
| Z1454310449 | C10H10N3F | 191.21 | Fc1c(CNc2c[nH]nc2)cccc1 |
| Z1587220559 | C14H13N3O | 239.28 | Oc1c(CNc2nc(cccc3)c3[nH]2)cccc1 |
| Z1623890017 | C11H13N3OS | 235.31 | C(CC1)CCN1c1n[o]c(-c2ccc[s]2)n1 |
| Z1741964527 | C16H17NO | 239.32 | OC(C1)CN1C(c1ccccc1)c1ccccc1 |
| Z1802166390 | C13H16NOCl | 237.73 | C[C@H](C1)[C@@H]1C(NCCc(cc1)ccc1Cl)=O |
| Z1891776064 | C9H7N3OCl2 | 244.08 | OCc1c[n](-c(cc2)cc(Cl)c2Cl)nn1 |
| Z19234337 | C10H9NO3 | 191.19 | COC(COc(cc1)ccc1C#N)=O |
| Z192955056 | C12H15N2O2F | 238.26 | COC(N(CC1)CCN1c(cc1)ccc1F)=O |
| Z1929757385 | C11H15N2OF | 210.25 | CC(C)(CN(C1)c(nccc2)c2F)C1O |
| Z1945710531 | C10H12N2O2S | 224.28 | CC(c1nc(-c2c(C)[o]nc2)c[s]1)OC |
| Z19731563 | C13H14N2O3 | 246.27 | Cc1cc(NC(COc2ccc(C)cc2)=O)n[o]1 |
| Z19735192 | C14H12NO2F | 245.26 | O=C(COc1ccccc1)Nc1cccc(F)c1 |
| Z19735981 | C12H15NO2 | 205.26 | O=C(COc1ccccc1)N1CCCC1 |
| Z19750454 | C12H12N2O2S | 248.31 | Cc1cc(NC(CSc2ccccc2)=O)n[o]1 |
| Z198195774 | C13H12N2O | 212.25 | NC(Nc(cc1)ccc1-c1ccccc1)=O |
| Z1992316287 | C8H12N2 | 136.20 | CCNc1ncccc1C |
| Z2204875953 | C10H8N2O2 | 188.19 | C1Oc(cc(cc2)-c3n[nH]cc3)c2O1 |
| Z2434225559 | HCl.C9H10N2O | 198.65 | NC(c(cc1)cc2c1NCC2)=O.Cl |
| Z2467208649 | C10H13N3 | 175.24 | CC(C)Nc1nc(cccc2)c2[nH]1 |
| Z26333434 | C15H11N3 | 233.28 | N#Cc1ccc(C[n]2c(cccc3)c3nc2)cc1 |
| Z26333448 | C14H11N2F | 226.26 | Fc1ccc(C[n]2c(cccc3)c3nc2)cc1 |
| Z26548228 | C15H18N2O | 242.32 | N#Cc1cccc(NC(CCC2CCCC2)=O)c1 |
| Z26794338 | C12H9N3O3 | 243.22 | O=C(c1ccc[o]1)Nc(cc1)cc(N2)c1NC2=O |
| Z2697514548 | C9H9N3O | 175.19 | C[n]1nc(C(N)=O)c2c1cccc2 |
| Z272156568 | C8H10N2O2 | 166.18 | CCOc(nccc1)c1C(N)=O |
| Z27678561 | C12H12N2O2S | 248.31 | COc(cc1)cc2c1nc(NC(C1CC1)=O)[s]2 |
| Z27797417 | C13H13NO2S | 247.32 | Cc1ccc(C(Nc(cccc2)c2OC)=O)[s]1 |
| Z27805986 | C14H11NOF2 | 247.25 | O=C(Cc(cc1)ccc1F)Nc(cc1)ccc1F |
| Z281077318 | C13H16N2O3 | 248.28 | COC(c(cccc1)c1NC(N1CCCC1)=O)=O |
| Z28226359 | C14H17NO3 | 247.30 | COC(c(cc1)ccc1NC(C1CCCC1)=O)=O |
| Z28290384 | C14H12NO2F | 245.26 | COc1cccc(C(Nc(cccc2)c2F)=O)c1 |
| Z28429411 | C14H15NOS | 245.35 | Cc1ccc(CNC(Cc2ccc[s]2)=O)cc1 |
| Z2856434783 | C14H14NF | 215.27 | Fc1ccc(CNCc2ccccc2)cc1 |
| Z285675722 | C12H13N3 | 199.26 | Cc1nc(Nc2ccccc2)nc(C)c1 |
| Z285782452 | C8H11NO2S | 185.25 | CNc(cccc1)c1S(C)(=O)=O |
| Z29077827 | C13H17N3O | 231.30 | CCC[n]1c(NC(CC)=O)nc2c1cccc2 |
| Z300245038 | C8H11N2O2FS | 218.25 | CN(Cc1cc(F)ccc1)S(N)(=O)=O |
| Z30162334 | C16H23NO | 245.37 | CC(C)C(N1CCC(Cc2ccccc2)CC1)=O |
| Z30891796 | C14H13NO2 | 227.27 | Cc1cc(OC(c2ccncc2)=O)c(C)cc1 |
| Z30917949 | C13H13N3O | 227.27 | CCc(cc1)ccc1NC(c1nccnc1)=O |
| Z31385861 | C13H14N2O | 214.27 | CC(C)NC(c1nc2ccccc2cc1)=O |
| Z31478129 | C10H13NO2 | 179.22 | CCOC(NCc1ccccc1)=O |
| Z31697001 | C9H10NO2Cl | 199.64 | CCOC(Nc1cccc(Cl)c1)=O |
| Z319545618 | C12H11N3 | 197.24 | Cc1ncc[n]1Cc1cccc(C#N)c1 |
| Z32665176 | C15H17NO2 | 243.31 | CC(CC1)CCN1C(c1cc(cccc2)c2[o]1)=O |
| Z328695024 | C10H15N5 | 205.26 | CC(C)N(C)c1ncnc2c1cn[n]2C |
| Z332370018 | C10H9N3O2 | 203.20 | CNC(C(N1)=Nc(cccc2)c2C1=O)=O |
| Z336089202 | C13H13NO | 199.25 | Nc(cccc1)c1OCc1ccccc1 |
| Z363993198 | C10H8N3Cl | 205.65 | Nc1nccc(-c(cc2)ccc2Cl)n1 |
| Z371866204 | C10H11N3O2S | 237.28 | CNC(Nc1nc(ccc(OC)c2)c2[s]1)=O |
| Z373221060 | C8H9N5 | 175.19 | C(C1)CN1c1ncnc2c1[nH]cn2 |
| Z384468096 | C14H19NO2 | 233.31 | CC(NCC1(CCOCC1)c1ccccc1)=O |
| Z419884046 | C16H23NO | 245.37 | CC(CC1)CCN1C(CCCc1ccccc1)=O |
| Z453319206 | C16H12N2O | 248.29 | O=C(c1ccccc1)Nc1cc2cccnc2cc1 |
| Z45516134 | C11H17NO2S | 227.33 | CCN(CC)S(c1ccc(C)cc1)(=O)=O |
| Z48847633 | C12H14N2S | 218.32 | Cc1c(-c2cc(C)c(C)cc2)nc(N)[s]1 |
| Z53834613 | C13H13N3O2 | 243.27 | Cc(cccc1)c1OCC(Nc1ncccn1)=O |
| Z55290386 | C13H11N2O2F | 246.24 | Oc1cccc(NC(Nc(cc2)ccc2F)=O)c1 |
| Z55671900 | C9H8O2F2 | 186.16 | CC(c(cc1)ccc1OC(F)F)=O |
| Z56767623 | C11H12N2OS | 220.30 | C(COCC1)N1c1nc(cccc2)c2[s]1 |
| Z56791867 | C10H15N5 | 205.26 | CCN(CC)c1cc(C)nc2ncn[n]12 |
| Z56837087 | C9H7N2OCl | 194.62 | N#CCC(Nc(cc1)ccc1Cl)=O |
| Z56923284 | C12H12N2O2S | 248.31 | Nc(cc1)ccc1S(Nc1ccccc1)(=O)=O |
| Z57101343 | C12H11N5 | 225.25 | Cc(cc1)ccc1Nc1ncnc2c1cn[nH]2 |
| Z57299526 | C12H11N3O | 213.24 | C(c1ccc[o]1)Nc1nc(cccc2)c2[nH]1 |
| Z57299529 | C15H15N3 | 237.31 | C(CNc1nc(cccc2)c2[nH]1)c1ccccc1 |
| Z57345491 | C12H17N2Cl.HCl | 261.20 | CC(CC1)CCN1c(ccc(N)c1)c1Cl.Cl |
| Z57515803 | C13H10NO2Cl | 247.68 | O=C(c1cnccc1)OCc(cc1)ccc1Cl |
| Z57792053 | C10H9N2OCl | 208.65 | CCc1nc(-c(cc2)ccc2Cl)n[o]1 |
| Z68195082 | C14H13NO3 | 243.26 | Oc(cccc1)c1NC(COc1ccccc1)=O |
| Z86622311 | C10H7N2O2Cl | 222.63 | O=C(c1cccc(Cl)c1)Nc1n[o]cc1 |
| Z915492990 | C10H11N3OS | 221.28 | C[n]1nccc1C(NCc1ccc[s]1)=O |
| Z933326822 | C13H13NO | 199.25 | Nc1cccc(OCc2ccccc2)c1 |
| Z933840894 | C9H10N2OClF | 216.64 | CNC(NCc(c(F)ccc1)c1Cl)=O |


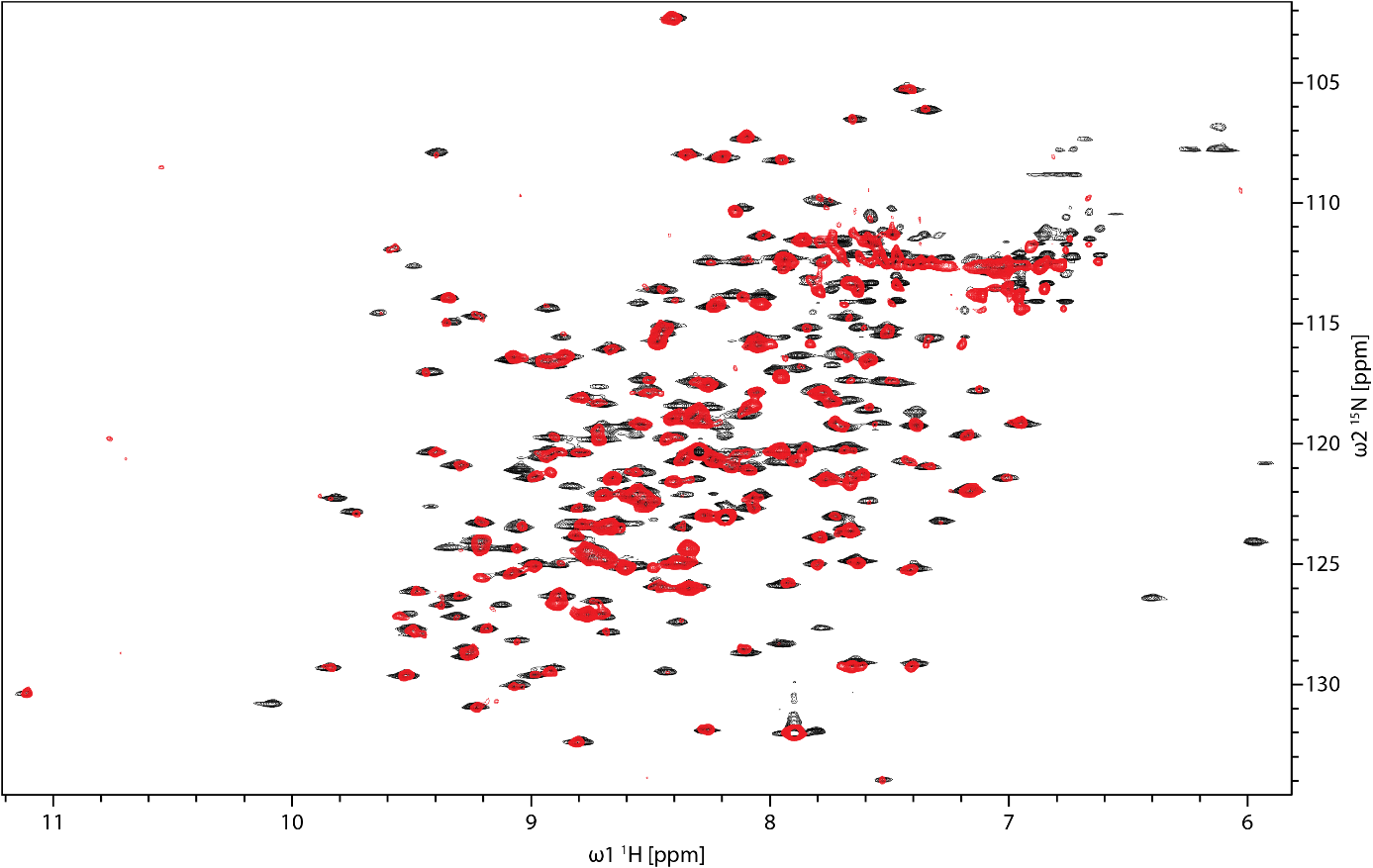
Supplementary figure 1: Superposition of two BEST-TROSY spectra of PLPro with and without 4 % DMSO in the buffer shown in red and black, respectively. Field strength varied between the two measurements: The spectrum with DMSO was measured at 600 MHz, the spectrum without DMSO was measured at 800 MHz. Protein concentration was sevenfold higher for the spectrum without DMSO (350 µM). The majority of the signals experience no or very minor shifts, meaning the fold of the protein is retained. Loss of low intensity signals in the spectrum with DMSO is due to the difference in protein concentration and field strength difference.


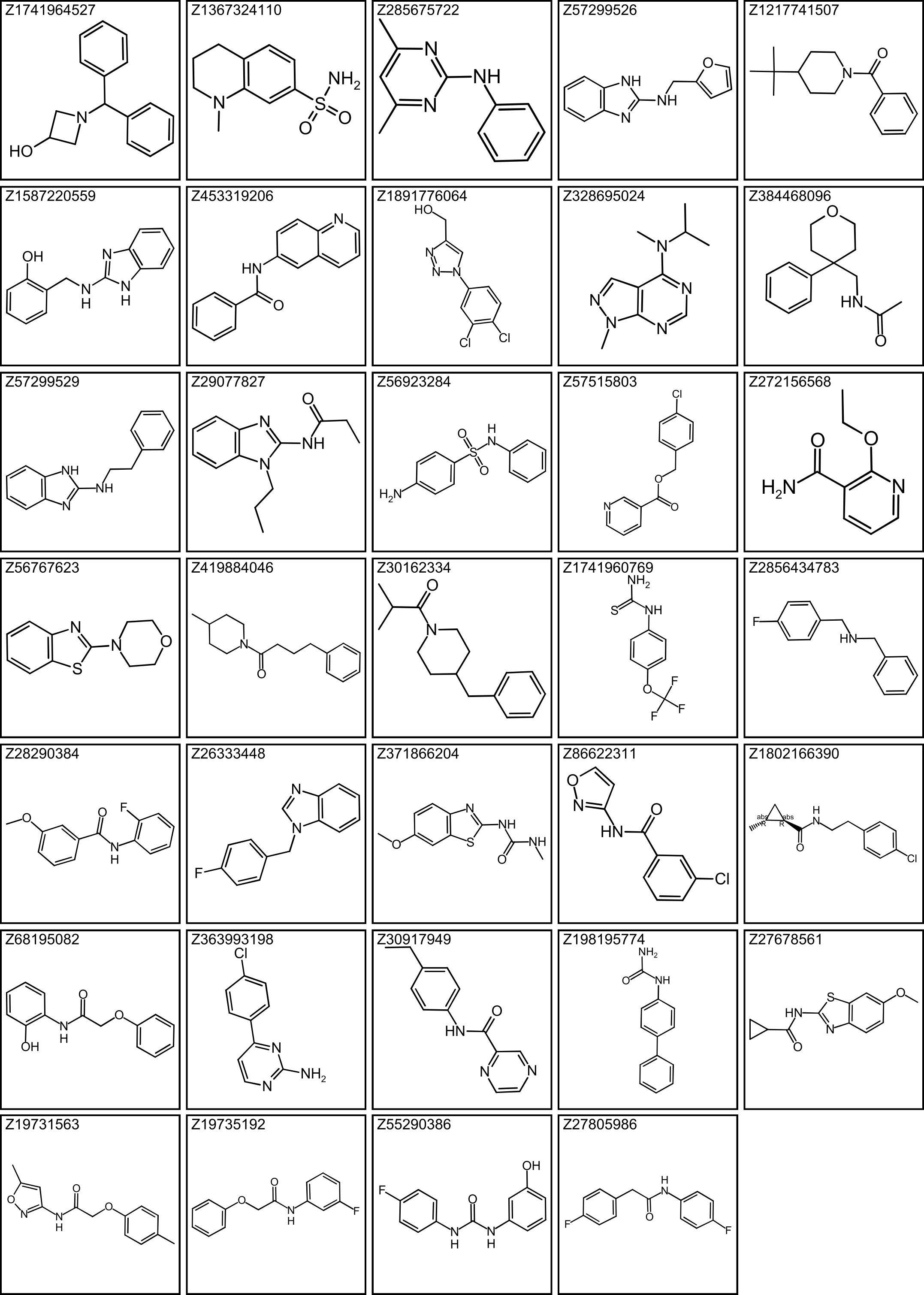
Supplementary figure 2: Molecular structures and identifiers of all 34 ligands that showed both WLOGSY and STD effects in the fragment-based screening.


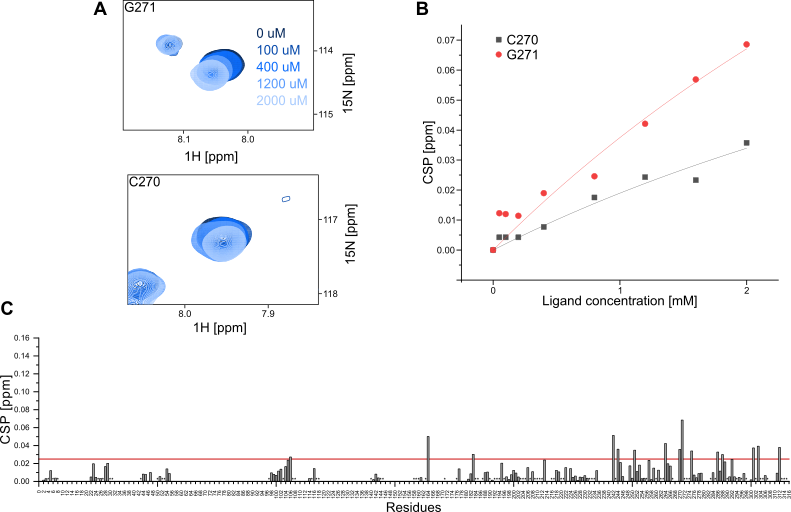


Supplementary figure 3: Analysis of compound Z1367324110. A: Two examples of shifting signals from the titration. Included signals are of residues C270 and G271. Included titration steps are 0 µM, 100 µM, 400 µM, 1200 µM and 2000 µM. B: CSPs at rising ligand concentrations plotted for selected amino acids and globally fitted for K_D_ estimation (7.4±6.5 mM). C: Bar plot of CSPs at 2000 µM for every residues. Stars indicate that the amino acid has an assignment but could not be plotted due to e.g. overlap with other signals.


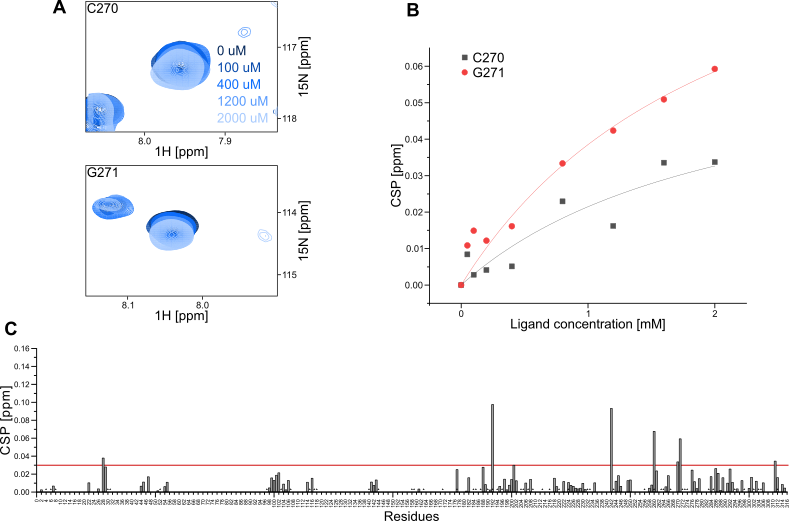


Supplementary figure 4: Analysis of compound Z1587220559. A: Two examples of shifting signals from the titration. Included signals are of residues C270 and G271. Included titration steps are 0 µM, 100 µM, 400 µM, 1200 µM and 2000 µM. B: CSPs at rising ligand concentrations plotted for selected amino acids and globally fitted for K_D_ estimation (2.1±1.1 mM). C: Bar plot of CSPs at 2000 µM for every residues. Stars indicate that the amino acid has an assignment but could not be plotted due to e.g. overlap with other signals.

Supplementary table 3: All Tanimoto coefficients between the five chosen screening compounds and known PLPro binders from the PDB.

| pdb ligand/screening compounds | Z57299529 | Z1217741507 | Z1367324110 | Z1587220559 | Z1891776064 |
| --- | --- | --- | --- | --- | --- |
| VIR250 (6WUU) | 0.118 | 0.086 | 0.066 | 0.116 | 0.058 |
| VIR251 (6WX4) | 0.08 | 0.075 | 0.064 | 0.079 | 0.045 |
| peptidomimetic inhibitor, based on VIR251 (8IHO) | 0.084 | 0.151 | 0.068 | 0.092 | 0.06 |
| GRL0617 (7CMD) | 0.071 | 0.101 | 0.074 | 0.081 | 0.053 |
| PLP_Snyder441 (7JN2) | 0.072 | 0.102 | 0.064 | 0.082 | 0.054 |
| PLP_Snyder630 (7SGV) | 0.091 | 0.141 | 0.045 | 0.101 | 0.084 |
| PLP_Snyder496 (7KOK) | 0.077 | 0.094 | 0.059 | 0.086 | 0.05 |
| PLP_Snyder530 (7JIV) | 0.075 | 0.092 | 0.058 | 0.084 | 0.049 |
| PLP_Snyder494 (7KOJ) | 0.08 | 0.085 | 0.054 | 0.088 | 0.055 |
| PLP_Snyder457 (7JIR) | 0.071 | 0.101 | 0.074 | 0.081 | 0.053 |
| PLP_Snyder495 (7JIT) | 0.064 | 0.1 | 0.066 | 0.072 | 0.047 |
| PLP_Snyder608 (7SGU) | 0.087 | 0.12 | 0.067 | 0.109 | 0.08 |
| XR8-89 (7LBR) | 0.075 | 0.106 | 0.053 | 0.082 | 0.054 |
| XR8-24 (7LBS) | 0.062 | 0.111 | 0.081 | 0.069 | 0.065 |
| XR8-83 (7LLF) | 0.075 | 0.106 | 0.053 | 0.082 | 0.054 |
| XR8-69 (7LLZ) | 0.056 | 0.106 | 0.058 | 0.072 | 0.059 |
| XR8-65 (7LOS) | 0.059 | 0.106 | 0.053 | 0.074 | 0.054 |
| Jun9-84-3 (7RZC) | 0.091 | 0.066 | 0.079 | 0.088 | 0.082 |
| Jun9-72-2 (7SQE) | 0.115 | 0.07 | 0.049 | 0.094 | 0.127 |
| Acriflavine (7NT4) | 0.048 | 0.093 | 0.069 | 0.079 | 0.071 |
| 4-(2-hydroxyethyl)phenol (7OFS) | 0.056 | 0.133 | 0.109 | 0.1 | 0.078 |
| p-hydroxybenzaldehyde (7OFT) | 0.07 | 0.104 | 0.086 | 0.106 | 0.062 |
| 3,4-Dihydroxybenzoic acid, methyl ester (7OFU) | 0.037 | 0.054 | 0.066 | 0.061 | 0.053 |
| N-(2-pyrrolidyl)-3,4,5-trihydroxybenzoylhydrazone (7QCG) | 0.06 | 0.065 | 0.076 | 0.096 | 0.092 |
| N-(3,5-dimethoxy-4-hydroxybenzyliden)thiosemicarbazone (7QCH) | 0.063 | 0.055 | 0.081 | 0.103 | 0.083 |
| N-(3,4-dihydroxybenzylidene)-thiosemicarbazone (7QCI) | 0.057 | 0.062 | 0.073 | 0.093 | 0.075 |
| N-(2,4-dihydroxybenzylidene)-thiosemicarbazone (7QCJ) | 0.047 | 0.078 | 0.089 | 0.095 | 0.063 |
| N-(2,5-dihydroxybenzylidene)-thiosemicarbazone (7QCK) | 0.118 | 0.066 | 0.064 | 0.14 | 0.054 |
| N-(3-methoxy-4-hydroxy-acetophenone)thiosemicarbazone (QCM) | 0.09 | 0.084 | 0.045 | 0.1 | 0.071 |
| methyl 4-{2-[3-(2-{[(1R)-1-(naphthalen-1-yl)ethyl]carbamoyl}phenyl)propanoyl]hydrazinyl}-4-oxobutanoate (8EUA) | 0.09 | 0.086 | 0.045 | 0.118 | 0.055 |
| remodilin NCGC 390004 (8G62) | 0.062 | 0.131 | 0.162 | 0.061 | 0.076 |
| compound S43 (7E35) | 0.088 | 0.172 | 0.082 | 0.096 | 0.073 |
| ebselen (7M1Y) | 0.139 | 0.172 | 0.054 | 0.091 | 0.056 |
| 3k (7TZJ) | 0.092 | 0.167 | 0.085 | 0.1 | 0.076 |
| YM155 (7D7L) | 0.087 | 0.093 | 0.09 | 0.085 | 0.07 |


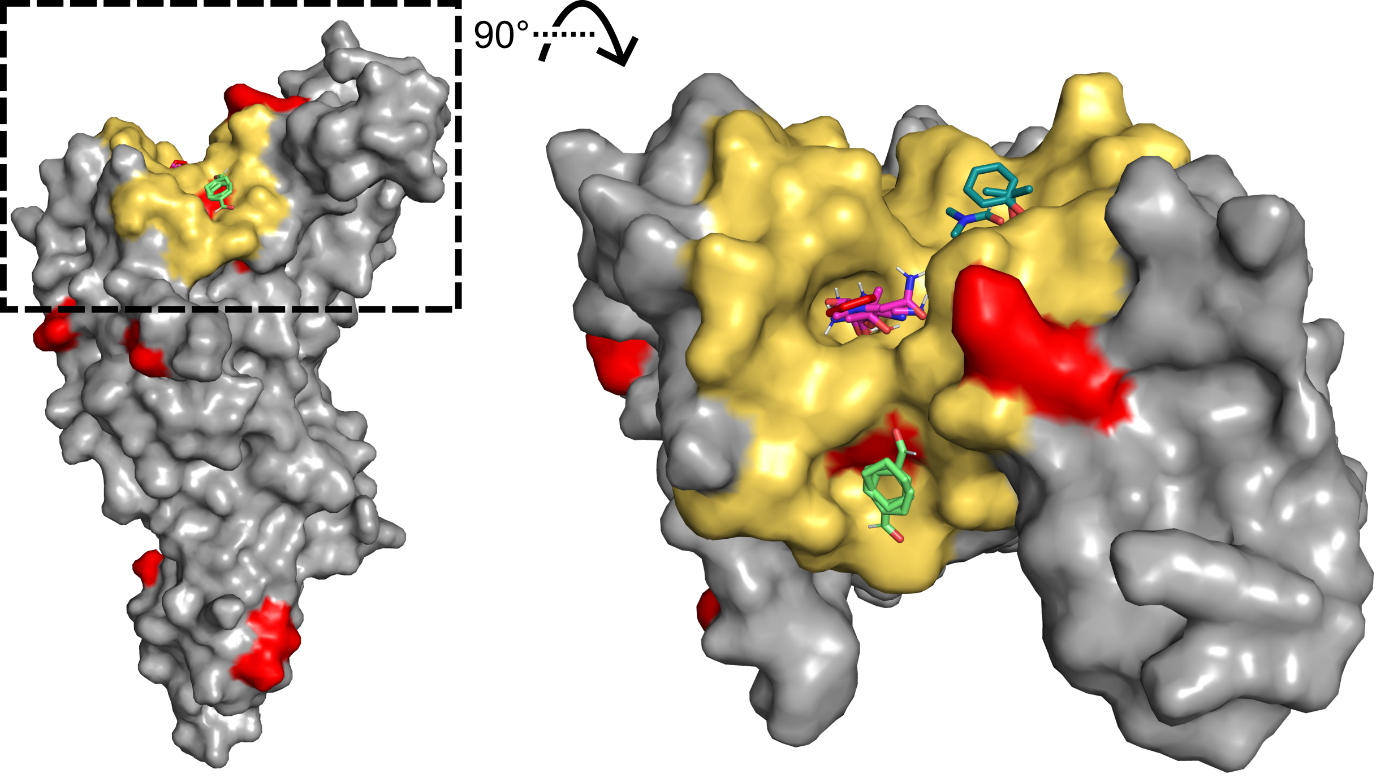


Supplementary figure 5: Additional cleft calculated by PDBsum shown in yellow on top of the protein. Relevant CSPs within the clefts are colored in red. Three cross-clusters obtained by the FTMap analysis are shown inside the cleft to indicate the possible binding sites of ligands.
